# The economic cost of tobacco smoking and secondhand smoke in Greece: Musculoskeletal disorders the leading contributor to smoking-related morbidity

**DOI:** 10.18332/tpc/113091

**Published:** 2019-11-15

**Authors:** Konstantina Koronaiou, Sofia Delipalla

**Affiliations:** 1 University of Macedonia, Thessaloniki, Greece

**Keywords:** smoking, secondhand smoke, economic cost, healthcare expenditure, mortality cost, morbidity cost

## Abstract

**INTRODUCTION:**

The high proportion of the population in Greece that is active and passive smoking makes smoking the leading risk factor for death and disability. Tobacco use creates a high cost to society and yet relevant research for Greece is limited.

**METHODS:**

The cost-of-illness approach is used to estimate the economic cost of smoking and, for the first time, of secondhand smoke (SHS) exposure in Greece. The analysis covers more health conditions, causally related to smoking, than those included in such studies.

**RESULTS:**

Based on data from the Global Burden of Diseases Study 2017, total economic cost of tobacco smoking and SHS exposure is estimated to be €7.2 billion in 2017, which is equivalent to almost 4% of GDP in Greece. SHS exposure accounts for 8.9% of total cost. Direct cost of smoking is slightly less than indirect cost. Indirect cost is relatively higher for males. Musculoskeletal disorders and diabetes are found to comprise the greatest fraction of morbidity cost of smoking and SHS exposure, respectively. Cardiovascular diseases are found to be the main cause of mortality costs for both smoking and SHS exposure.

**CONCLUSIONS:**

Total economic cost of tobacco smoking and SHS exposure in Greece is estimated to be more than double the revenue from tobacco taxes. Smoking imposes a heavy economic burden, underlining the need for efficient interventions, including effective implementation and enforcement of existing anti-tobacco policies.

## INTRODUCTION

The high proportion of smoking and secondhand smoke (SHS) exposure makes tobacco use the leading risk factor for death and disability in Greece^1^. The age standardized prevalence of current tobacco smokers is among the highest in the world (43.7%), ranking Greece third globally and second among the EU countries^2^. Moreover, in 2014, 64.2% of people aged ≥15 years were exposed daily to tobacco smoke indoors, whilst the EU average was only 21.6%^3^.

Research on the economic cost of smoking in Greece, however, is limited. Tsalapati et al.^4^ estimated the hospital costs for the treatment of smoking-attributable diseases and found them to be €554 million in 2011. In this study, which focuses on the calculation of the direct cost of active smoking, the annual cost approach (prevalencebased) was used and smoking attributable fractions for each disease were calculated using estimated relative risks of mortality from the American Cancer Society’s Prevention Study^5^. There are only a couple of studies that estimate both the direct and indirect costs of smoking and none focuses exclusively on Greece. Goodchild et al.^6^, in a study on global economic cost of smoking, found that the cost in Greece was €4.7 billion, equivalent to 2.4% of Gross Domestic Product (GDP) in 2012. Jarvis et al.^7-8^, a study commissioned by the DG SANCO for the EU, estimated it at €6.2 billion in 2000 and €11.2 billion in 2009, corresponding to 4.5% of GDP. Results differ mainly due to differences in mortality cost estimation. Jarvis et al.^7-8^ use the willingness-to-pay approach while Goodchild et al.^6^ employed the human-capital approach, with the former producing much higher estimates. Both studies cover only a certain number of diseases, such as cancers, cardiovascular and respiratory diseases, with Goodchild et al.^6^ also including tuberculosis and lower respiratory infections. Moreover, they do not estimate the economic cost of SHS, which for Greece is expected to be relatively high. Jarvis et al.^7-8^ estimated only part of the direct cost of SHS that of public healthcare spending.

In general, four main diseases dominate in the estimation of mortality and morbidity costs due to non-communicable diseases: cardiovascular diseases, diabetes, cancer, and chronic respiratory diseases. A number of key diseases, not included in the prevailing list such as musculoskeletal diseases, impose substantial social and private costs^9^. There exist a number of studies relating musculoskeletal disorders to smoking^10-13^. The most known adverse effects of smoking are the loss of bone mineral content and increased incidence of fractures^14^ and back pain^15^. Avoiding smoking is one of the strategies that the European Action towards Better Musculoskeletal Health recommends for the prevention and management of musculoskeletal disorders^16^. Musculoskeletal conditions are the leading cause of work absenteeism and disability, and hence impose a significant economic cost to society through lost productivity and increased healthcare spending^17^.

The present study’s contribution is twofold. First, it presents the most detailed and systematic study on the economic burden of tobacco smoking and passive smoking in Greece to date, and estimates for the first time the economic cost of secondhand smoke in the country. Second, it contributes to the global research of economic cost of smoking by covering more health conditions, whose causal relationship to smoking has been established, than those included in previous studies, demonstrating for the first time, to the best of our knowledge, that the main contributor to productivity loss due to smoking is musculoskeletal disorders.

## METHODS

The study adopts the cost-of-illness approach^18-21^. The economic cost of smoking is divided into direct cost, health care for which payments are made, and indirect cost, productivity losses due to morbidity and early mortality from smoking-related diseases. Non-healthcare costs, such as transportation to healthcare providers, informal care, property losses from fires caused by smoking etc. and an imputed value for lost household production services are not included due to lack of data, as in most studies. The population of interest is males and females aged 30–34 years (health consequences appear after some years of exposure) to 65–69 years (representing the age of retirement).

The methodology used is based on the WHO toolkit on the economic cost of smoking^21^ and on Goodchild et al.^6^. The smoking attributable healthcare expenditure (SAE), by financing source *s* and type of healthcare service *k*, is calculated from the formula:

$${\mathrm{SAE}}_{\mathrm{sk}}=\mathrm{SAF}\times {\mathrm{THE}}_{\mathrm{sk}}$$ 1

where SAF is the smoking attributable fraction and THE is total national annual expenditures by financing source *s* (i.e. public health expenditures, private insurance, private payments and the rest of the world financing schemes for non-residents) and type of healthcare service *k* (i.e. inpatient care, outpatient care, medication and other services such as ancillary services and preventive care).

The smoking attributable indirect morbidity cost (SAIC), caused by disease *i* among population subgroup *j*, is calculated from:

$${\mathrm{SAIC}}_{\mathrm{ij}}={\mathrm{SAF}}_{\mathrm{ij}}\times {\mathrm{EMP}}_{\text{j}}\times {\mathrm{YLD}}_{\mathrm{ij}}\times \mathrm{PROD}$$ 2

where SAF is smoking attributable fraction of indirect morbidity cost, EMP is employment to population ratio, YLD is number of years lost to disability, and PROD is GDP per worker.

Mortality cost represents the value of lost productivity due to lives of working age lost prematurely from smoking related diseases. The value of life lost is quantified using the human-capital approach, which values life according to loss of foregone market earnings^21^. The smoking-attributable mortality cost (SAMC) resulting from dying from disease *i* among population subgroup *j* is calculated from:

$${\mathrm{SAMC}}_{\mathrm{ij}}={\mathrm{SAF}}_{\mathrm{ij}}\times \sum_{a=\mathrm{mina}}^{\mathrm{maxa}}\;(\text{TDEAT}{\text{H}}_{\text{ija}}\times \text{PVL}{\text{E}}_{\text{ja}})$$ 3

where, SAF is smoking-attributable fraction of death, TDEATH is total number of deaths, PVLE is total discounted present value of lifetime earnings, and mina and maxa are minimum and maximum age groups, respectively.

The PVLE is calculated, using the approach developed by Max et al.^22^, from:

$${\text{PVLE}}_{\text{ag}}=\sum_{\text{n}=\text{a}}^{\text{max}}\;(\text{SUR}{\text{V}}_{\text{ag}}(\text{n}))\times [\text{EM}{\text{P}}_{\text{g}}(\text{n})\times \mathrm{PROD}]\times {\left(1+\text{V}\right)}^{\text{n}-\text{a}}/{\left(1+\text{r}\right)}^{\text{n}-\text{a}}$$ 4

where, PVLE is present discounted value of lifetime earnings for a person of age *a* and gender *g*, SURV is the probability that a person of age *a* and gender *g* will survive to age *n*, *a* is the age of the person at present (death), *g* is gender of the person, EMP is the proportion of population of gender *g* and age *n* that are employed , V is growth rate of labor productivity, and r the discount rate. Following a standard practice, we assumed a 1% labor productivity growth rate. We assumed no discounting for human life, but performed a sensitivity analysis with a 3% discount rate.

The list of diseases included in the estimation of morbidity and mortality costs is given in Table 1. Data used are for 2017, apart from life tables which are for 2016. Due to lack of national data by smoking status, we used estimated SAFs by disease, age and gender from the 2017 Global Burden of Disease Study^23^. SAFs for tobacco smoking and SHS by disease and gender for people aged 30–69 years are given in Table 2. These SAFs are higher than the corresponding SAFs for people of all ages. For example, SAF for all deaths related to smoking is 40.8% for males aged 30–69 years, while for the whole male population the corresponding SAF is 29.2%.

**Table 1:** List of diseases for which smoking-attributable mortality and morbidity costs were estimated

**Communicable diseases**
***Respiratory infections and tuberculosis:*** Tuberculosis, Lower respiratory infections (√)
***Non-communicable diseases***
***Neoplasms:*** Esophageal, Stomach, Liver, Larynx, ‘Tracheal, bronchus, and lung’ (√), Breast (√), Cervical, Prostate, ‘Colon and rectum’, ‘Lip and oral cavity’, Nasopharynx, Other pharynx, Pancreatic, Kidney, Bladder, Leukemia
***Cardiovascular diseases:*** Ischemic heart disease (√), Stroke (√), Atrial fibrillation and flutter, Aortic aneurysm, Peripheral artery disease
***Chronic respiratory diseases:*** Chronic obstructive pulmonary disease (√), Asthma
***Digestive diseases:*** Upper digestive system diseases, Gallbladder and biliary diseases
***Neurological disorders:*** Alzheimer’s disease and other dementias, Parkinson’s disease, Multiple sclerosis
***Diabetes and kidney diseases:*** Diabetes mellitus (√)
***Musculoskeletal disorders:*** Rheumatoid arthritis, Low back pain
**Injuries**
***Transport injuries, Unintentional injuries, Self-harm and interpersonal violence***

Morbidity and mortality costs related to secondhand smoke were estimated only for marked (√) diseases.

**Table 2:** SAFs for smoking and SHS by disease and gender, people aged 30-69 years

	*Smoking*		*SHS*	
	*Males %*	*Females %*	*Males %*	*Females %*
**All causes**	40.82	27.57	3.59	3.22
**Communicable diseases**	36.53	26.74	7.23	9.02
Respiratory infections and tuberculosis	46.68	33.42	9.24	11.27
Tuberculosis	45.98	33.56	-	-
Lower respiratory infections	46.93	33.53	10.07	11.99
**Non-communicable diseases**	44.83	29.03	3.85	3.24
Neoplasms	52.16	26.54	2.69	1.99
Esophageal cancer	62.25	47.45	-	-
Stomach cancer	34.82	22.80	-	-
Liver cancer	32.99	22.54	-	-
Larynx cancer	86.69	74.70	-	-
Tracheal, bronchus, and lung cancer	85.44	75.11	6.66	8.00
Breast cancer	-	10.78	-	2.63
Cervical cancer	-	47.43	-	-
Prostate cancer	12.07	-	-	-
Colon and rectum cancer	28.25	19.93	-	-
Lip and oral cavity cancer	59.76	49.01	-	-
Nasopharynx cancer	46.92	33.71	-	-
Other pharynx cancer	71.94	63.50	-	-
Pancreatic cancer	37.20	41.83	-	-
Kidney cancer	33.66	23.29	-	-
Bladder cancer	60.56	45.65	-	-
Leukemia	47.10	35.75	-	-
Cardiovascular diseases	46.46	41.28	5.84	6.16
Ischemic heart disease	51.65	52.00	7.29	8.46
Stroke	41.16	38.66	4.56	5.51
Atrial fibrillation and flutter	30.43	20.07	-	-
Aortic aneurysm	74.89	61.28	-	-
Peripheral artery disease	71.38	55.85	-	-
Chronic respiratory diseases	62.17	49.08	8.37	9.45
Chronic obstructive pulmonary disease	71.60	58.63	9.75	11.59
Asthma	37.64	27.50	-	-
Digestive diseases	4.58	4.01	-	-
Neurological disorders	18.46	18.37	-	-
Diabetes mellitus	20.11	12.31	7.14	8.22
Musculoskeletal disorders	6.83	3.82	-	-
**Injuries**	0.97	1.09	-	-
Transport injuries	1.56	1.51	-	-
Unintentional injuries	1.00	1.23	-	-
Self-harm and interpersonal violence	0.03	0.10	-	-

a) SAFs were calculated based on data on deaths from IHME21. Estimated SAFs by IHME are available by 5-year age groups and for all ages. b) For the estimation of economic cost of smoking and secondhand smoke, authors used the estimated SAFs by IHME which are by 5-year age groups, gender and disease and not those reported here.

According to a report by GBD 2017 Risk Factor Collaborators^24^, these SAFs were determined using relative risk, exposure and the theoretical minimum risk exposure level (TMREL). Relative risk (RR) measures the strength of the association between the risk of developing a disease and exposure to a given factor, which in our case is tobacco smoking and passive smoking. RR estimation was based on data collected from randomized controlled trials, cohort, pooled cohort, and case-control studies.

With regard to exposure to tobacco smoking, it is taken to be the prevalence of current use and the prevalence of former use of any smoked tobacco product. Exposure among current smokers is estimated using cigarettes per smoker per day or pack-years, while exposure among former smokers is estimated using years since quitting. On the other hand, the exposure to SHS is the average daily exposure to fine particulate matter (aerosols) from SHS with an aerodynamic diameter ≤2.5 μm (PM_2.5_), with concentration measured in μg/m^3^, among nonsmokers (this includes ex-smokers and occasional smokers). Making the assumption that all persons living with a daily smoker are exposed to tobacco smoke, the proportion of non-smokers who live with at least one smoker is calculated using unit record data on household composition. The GBD team also uses surveys to estimate the proportion of individuals exposed to secondhand smoke at work.

Finally, the theoretical minimum-risk exposure level for smoking is ‘all individuals are lifelong non-smokers’ and for secondhand smoke it is ‘zero exposure among non-smokers’, meaning that the non-smokers do not live with primary smokers.

Number of years lost to disability and number of deaths are also taken from the IHME^23^. Ratio of employment to population is from the International Labour Organization^25^ based on the EU Labour Force Survey. GDP is from the April 2019 World Economic Outlook database^26^. Number of workers and total national annual expenditures are from the Hellenic Statistics Authority (ELSTAT)^27^. Life tables are from the World Health Organization^28^.

## RESULTS

The total economic cost of smoking and SHS is estimated at €7.2 billion for Greece, which is equivalent to 3.99% of its GDP (Table 3). From this, €6.6 billion is attributable to smoking while about 9% is from SHS exposure.

**Table 3:** The economic cost of smoking and exposure to smoke, Greece, 2017

		*Direct Cost*	*Indirect Cost*			*Overall Cost*
			*Total*	*Morbidity*	*Mortality*	
**Smoking**	€ (million)	2941	3615	2227	1387	6555
	per capita €	273	336	207	129	609
	% total cost	44.9	55.1	34.0	21.2	100.0
**SHS**	€ (million)	324	317	167	150	642
	% total cost	50.6	49.4	26.1	23.4	100.0
**Smoking & SHS**	€ (million)	3265	3932	2394	1537	7197
	% total cost	45.4	54.6	33.3	21.4	100.0
	per capita €	303	365	222	143	668
	% GDP	1.81	2.18	1.33	0.85	3.99

Estimations based on IHME, ELSTAT, ILO & IMF data.

Interestingly, the total economic cost of tobacco smoking and SHS exposure is found to be more than double the revenue from tobacco taxes. In 2017, the revenues from excise and VAT on tobacco products were €2.1 billion and €0.6 billion, respectively^29^.

### Economic cost of smoking

The economic cost of smoking alone (€6.6 billion) corresponds to €608.8 per capita, equivalent to 3.64% of GDP; 20.29% of healthcare expenditure is related to smoking, which means that the direct cost is estimated at €2.94 billion. Calculations based on data from the IHME^25^ and ELSTAT^29^ show that the highest percentage of direct cost is due to public health expenditure (60.8%), followed by private health expenditure (39%), which is mainly out-ofpocket. Based on the distribution by type of healthcare service, 44.6% of direct cost is due to inpatient care, 37.6% due to medications and other services, and the rest is due to outpatient care.

**Figure 1:**
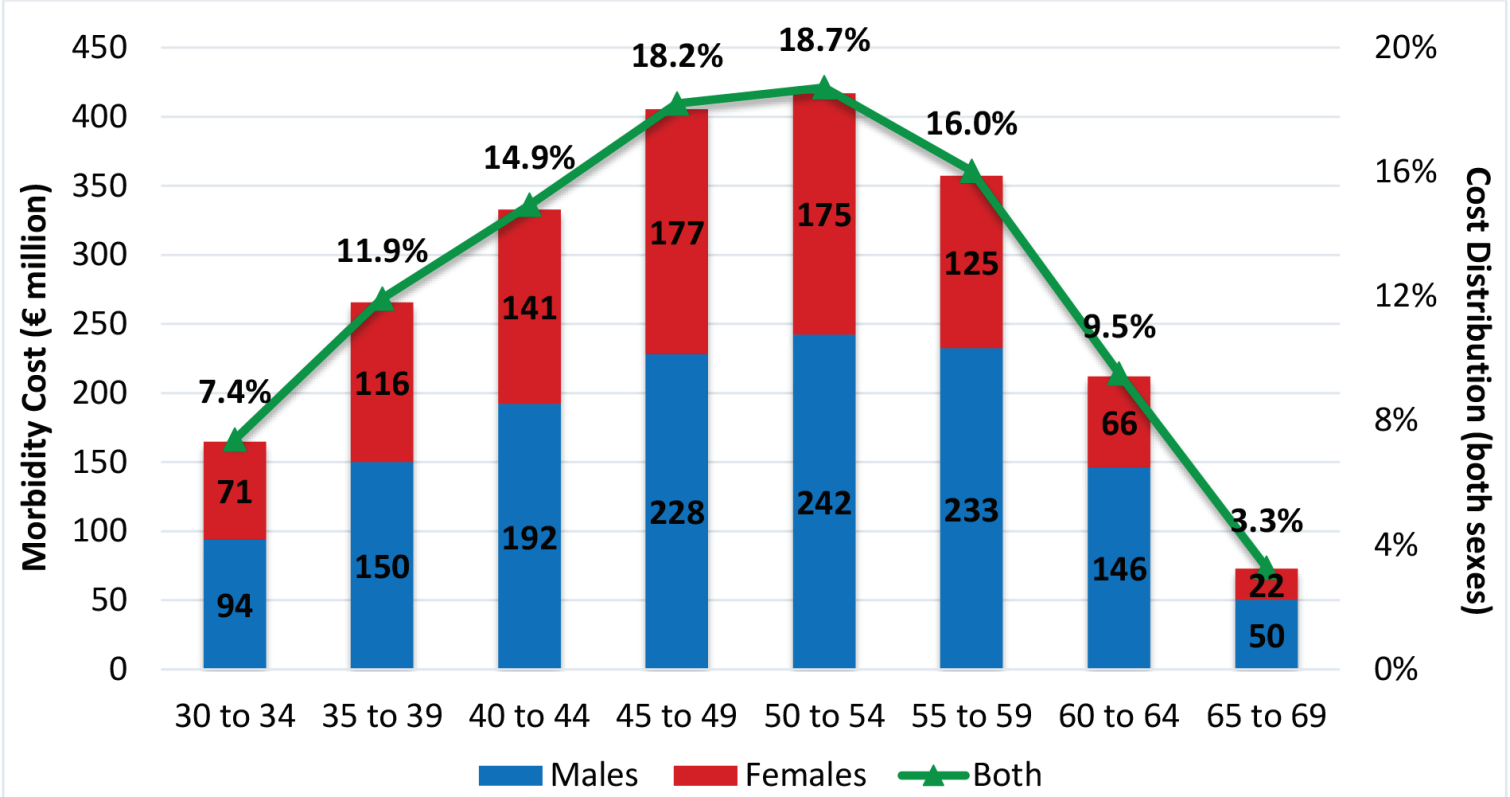
Smoking Attributable Morbidity Cost by gender / age

The indirect cost, estimated about €3.6 billion, accounts for 55.1% of total cost of smoking. Morbidity cost is 61.6% of indirect cost. The highest proportion (67.9%) of smoking-attributable morbidity cost results from working adults aged 40–59 years (Figure 1). After the age of 50 years, cost starts declining as both ratio of employment to population and smoking prevalence decrease in the older age groups. For all age groups, the highest proportion of morbidity cost results from males, and this proportion increases for the older age groups. Overall, almost 40% of smoking-attributable morbidity costs are caused by females.

The main contributor to morbidity cost is musculoskeletal disorders (mainly low back pain) accounting for 56.95% of morbidity costs (53.66% for males; 61.89% for females). The other main causes are chronic respiratory diseases (21.48% for males; 19.44% for females), diabetes (11.34% for males; 7.17% for females), and cardiovascular diseases (8.18% for males; 6.93% for females), as noted in Table 4. The proportion of morbidity cost generated by musculoskeletal disorders is higher at younger ages. As age increases, the relevant importance of musculoskeletal disorders decreases while mainly that of chronic respiratory diseases increases; a pattern followed by both genders.

**Table 4:** Morbidity and Mortality cost distribution by main causes, both genders

	*Cost of Smoking*		*Cost of SHS*	
	*Morbidity %*	*Mortality %*	*Morbidity %*	*Mortality %*
**Communicable diseases**	0.08	2.48	0.09	5.07
Respiratory infections & tuberculosis	0.08	2.48	0.09	5.07
**Non-communicable diseases**	98.56	97.19	99.91	94.93
Neoplasms	2.55	41.41	1.77	23.57
Cardiovascular diseases	7.68	51.55	5.02	66.09
Chronic respiratory diseases	20.66	2.45	40.21	3.82
Digestive diseases	0.22	0.54	-	-
Neurological disorders	0.57	0.83	-	-
Diabetes mellitus	9.67	0.39	52.90	1.45
Musculoskeletal disorders	56.95	0.01	-	-
Sense organ diseases	0.25	-	-	-
**Injuries**	1.36	0.33	-	-
Transport injuries	0.35	0.24	-	-
Unintentional injuries	1.00	0.08	-	-
Self-harm and interpersonal violence	0.01	0.00	-	-

Estimations based on IHME, ELSTAT, ILO & IMF data.

**Figure 2:**
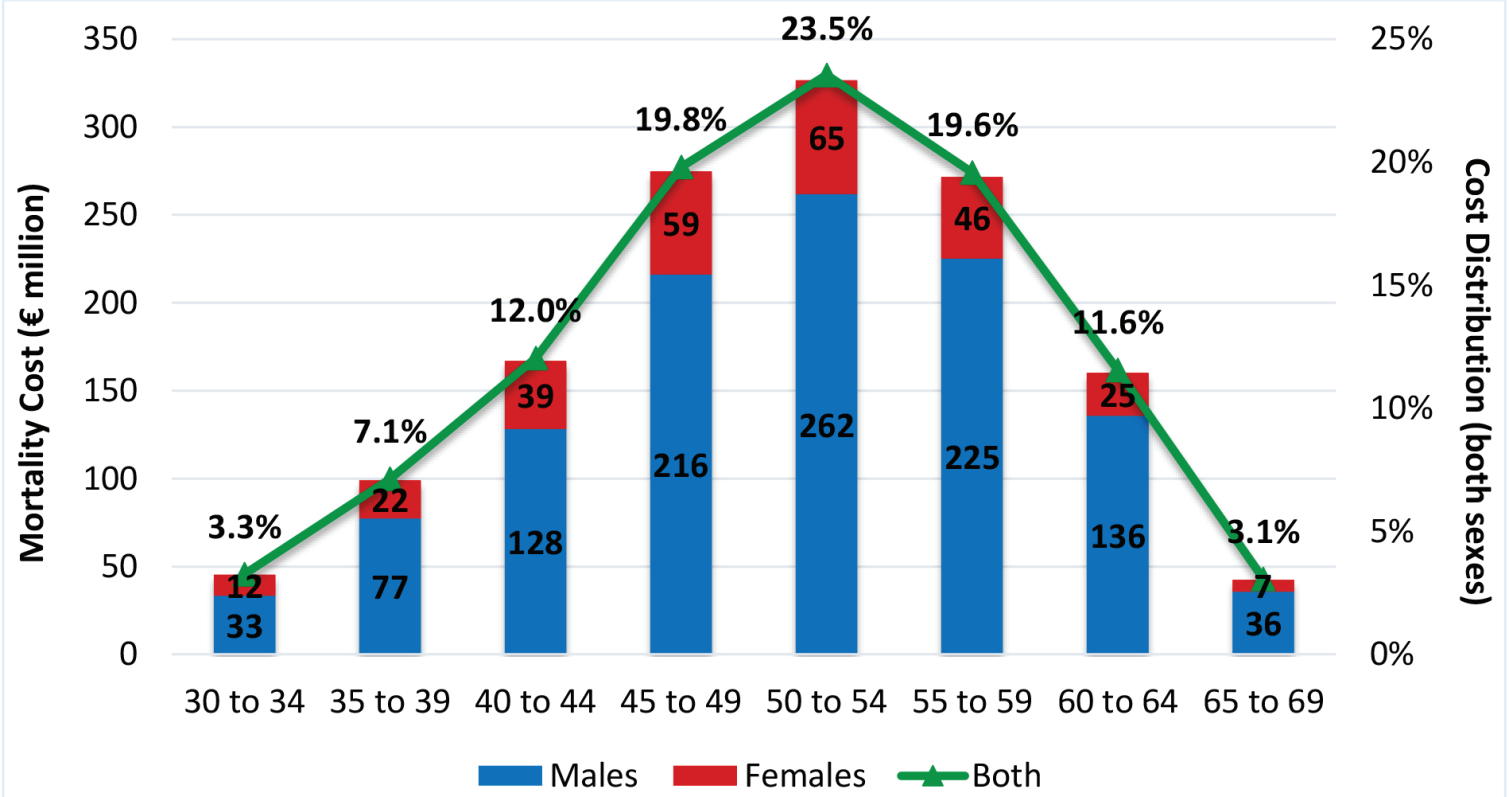
Smoking Attributable Mortality Cost by gender / age

Mortality cost reaches its peak for the age group 50–54 years. The highest proportion (62.9%) is due to working adults aged 45–59 years. For all age groups, the highest proportion results from males and increases with age (Figure 2). Overall, almost 20% of smoking-attributable mortality costs are caused by females.

The primary cause among males is cardiovascular diseases (mainly ischemic heart disease) accounting for 53.42% of mortality cost, followed by neoplasms (39.97%). For females, the main cause is neoplasms (47.26%) followed by cardiovascular diseases (43.95%). The proportion of mortality cost generated by cardiovascular diseases is higher at younger ages. As age increases, the relevant importance of cardiovascular diseases decreases while mostly that of neoplasms increases. This pattern is slightly different for females as the relevant importance of cardiovascular diseases increases for the age group 60–64 years and then declines again, but the variation is not great.

### Economic cost of exposure to SHS

**Figure 3:**
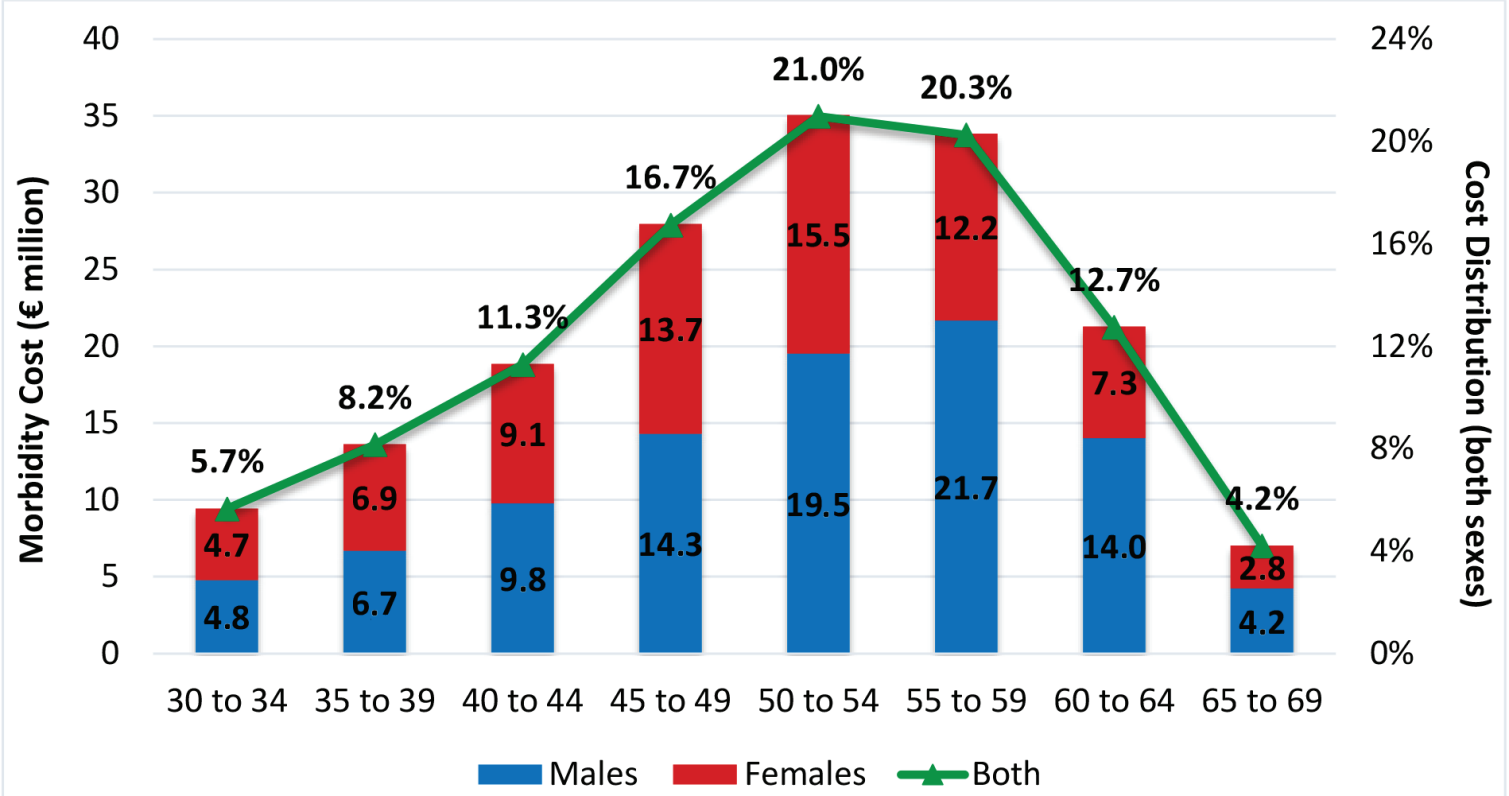
Morbidity Cost related to SHS by gender / age

Health care expenditure related to SHS exposure is €324.45 million (2.24% of total health care expenditure) and accounts for 50.57% of total cost of SHS exposure. Morbidity cost is €167.14 million and the highest proportion (58.0%) results from working adults aged 45–59 years (Figure 3). Among the younger age groups, the proportion of cost resulting from either gender is similar. Past the age of 50 years, however, the highest proportion of morbidity cost results from males. Overall, 43.2% of morbidity cost related to SHS exposure is caused by females, a proportion higher than that related to active smoking. Almost all morbidity cost due to SHS exposure is attributed to diabetes (55.34% for males; 49.69% for females) and chronic respiratory diseases (38.80% for males; 42.07% for females). The proportion of morbidity cost generated by diabetes is higher at younger ages.

**Figure 4:**
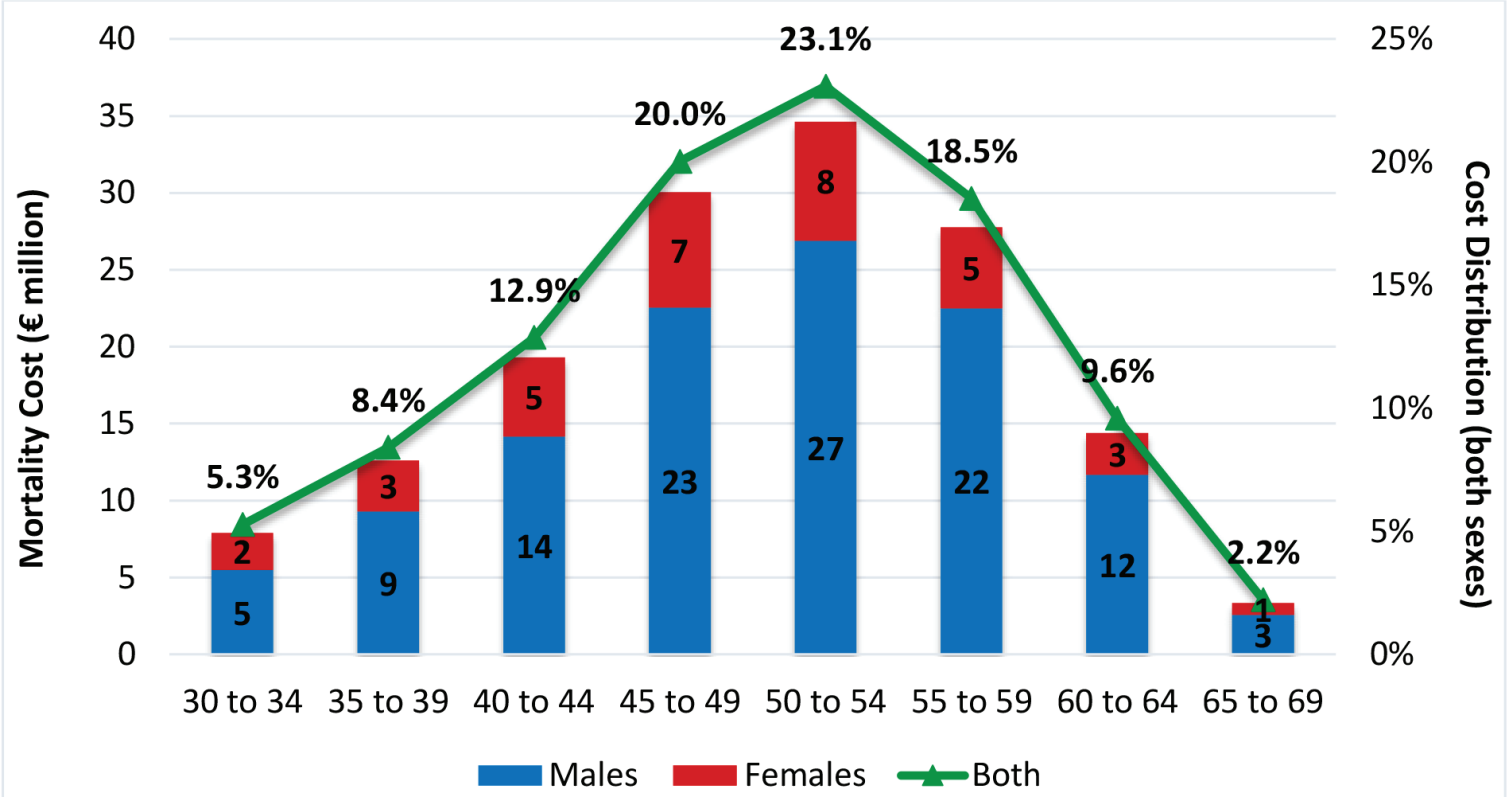
Mortality Cost related to SHS by gender / age

Mortality cost is €149.95 million and accounts for 23.37% of total SHS exposure cost. The highest proportion (61.6%) results from working adults aged 45–59 years (Figure 4). In all age groups, the highest proportion of mortality cost results from male exposure, ranging from 69.3% for the age group 30–34 years to 81.1% for the age group 60–64 years. Cardiovascular diseases (69.82% for males; 53.74% for females) and neoplasms (20.93% for males; 32.29% for females) are the main causes, responsible for 89.66% of mortality cost due to SHS. The proportion of mortality cost generated by cardiovascular diseases is higher for the two youngest age groups. As age increases, the relevant importance of cardiovascular diseases decreases and that of neoplasms increases.

### Sensitivity analysis

In our primary analysis, mortality cost was estimated following the most recent view that human life should not be discounted. However, up until recently, a standard practice in health economics was to assume a 3% discount rate. In this case, mortality cost of active and passive smoking is lower by 16.4% and 17.4%, respectively. As a result, total cost of smoking and SHS is lower by 3.5% and 4.1%, respectively.

## DISCUSSION

This study presents a systematic and detailed estimation of the economic cost of smoking and SHS exposure in Greece. Tobacco use is the leading risk factor for deaths and disability, with years of healthy life lost due to disability and early death attributed to tobacco (DALYs) as well as death rates higher than the corresponding average for the European region, high income countries or globally.

The total economic cost of tobacco smoking and passive smoking is estimated at €7.2 billion in 2017, an amount equivalent to 3.99% of GDP. From this amount, €6.6 billion is attributable to active and €642 million to passive smoking. More than half of morbidity cost is estimated to be caused by musculoskeletal disorders in the case of active smoking and by diabetes in the case of SHS. These findings might seem surprising but there are a number of studies linking musculoskeletal disorders with smoking^10-13^ and diabetes with SHS^30-31^. The greatest fraction of mortality cost, for both active smoking and SHS, is caused by cardiovascular diseases (51.6% and 66.1%, respectively). It may help to mention that SAFs might be high for some diseases but their contribution to the economic cost might be low if the number of deaths or years lost due to disability are relatively low compared to other diseases. Such an example is larynx cancer where 86.7% of deaths of males aged 30–69 years is related to tobacco smoking but the corresponding deaths for this group from larynx cancer is only 0.92% of all deaths.

These results of economic cost of smoking are not comparable with previous studies (Goodchild et al.^6^ and Jarvis et al.^7-8^) as there are differences in methodology, data and the number of diseases covered. Our approach is closer to that of Goodchild et al.^6^ but we use different data sources with more recent data and different discount rate for the estimation of mortality cost. However, our major difference is that we have included more tobacco-related diseases. One is musculoskeletal disorder, which was found to be the main cause of smoking-related morbidity cost. With regard to the economic cost of SHS, as far as we know, there is no previous research on this. Only Jarvis et al.^7-8^ estimated the public healthcare spending attributable to SHS, which is just a part of the direct cost of SHS.

### Limitations

The extent of the analysis was constrained by data limitations. Data on healthcare expenditure were not available by disease, gender and age, and hence we could not conduct a more detailed analysis of the direct cost. Moreover, SAFs were IHME estimations based on mortality data, and this might have led to an underestimation or overestimation of the direct cost. Even though all main costs have been taken into account, we did not have any data on non-health care costs or the value of lost household productivity. With regard to SHS, studies have shown that exposure to passive smoking increases the risk of asthma^32^. However, data on this are limited for Greece and, thereby, the cost of SHS might have been underestimated.

Finally, in estimating the economic cost of tobacco smoking and SHS separately, we may have overestimated the total cost as, given the data constraints, we did not take into account a possible correlation between them. Correlations among different risk factors are taken into consideration only for the general category ‘tobacco’, which includes smoking, SHS and chewing tobacco. As a check, we did calculate the economic cost of tobacco use based on data of the general category ‘tobacco’ and found that the cost differs only slightly from the estimates provided here.

### Policy implications

Our results have significant policy implications for tobacco control and public health. For brevity, we focus on two policy issues: complete implementation of anti-smoking legislation (WHO FCTC Article 8) and protection of policy making from tobacco industry influence (WHO FCTC Article 5.3)^33^.

The smoke-free law is the most implemented article of FCTC worldwide^34^. Reporting of exposure to tobacco smoke is extremely high in bars (87%) and restaurants (78%) while at the same time the EU averages are 20% and 9%, respectively^35^. It appears that the problem is a lack of political will to enforce smoke-free legislation. Contributing factors are the financial crisis, the fear of political cost but also the influence of the tobacco industry^36^. In their effort to attract foreign investment, all governments welcomed and embraced the idea of Greece becoming an epicentre of the tobacco industry’s investment. This is in violation of Article 5.3 of the WHO FCTC, which explicitly seeks to protect policy making from commercial and other vested interests of the tobacco industry in accordance with national legislation. Respecting the human right to clean air, Greek governments should implement effective smoke-free legislation and adopt all FCTC provisions to achieve significant positive results for public health and public budget.

## CONCLUSIONS

A high proportion of the population of Greece is active and passive smoking. According to our findings, the economic cost of tobacco smoking and SHS exposure is €7.2 billion (3.99% of GDP) in 2017, estimated to be more than double the revenue from tobacco taxes. From this amount, about 9% is caused by SHS. Our major finding is that musculoskeletal disorders are the main cause of smoking attributable morbidity cost, when they are added to the list of tobacco related diseases. It is clear that, apart from the unintended consequences of smoking on health, smoking creates a huge economic burden on society.
